# The Performance of Serum Alpha-Fetoprotein for Detecting Early-Stage Hepatocellular Carcinoma Is Influenced by Antiviral Therapy and Serum Aspartate Aminotransferase: A Study in a Large Cohort of Hepatitis B Virus-Infected Patients

**DOI:** 10.3390/v14081669

**Published:** 2022-07-29

**Authors:** Xiangjun Qian, Yanna Liu, Fengping Wu, Siyu Zhang, Jiao Gong, Yuemin Nan, Bo Hu, Junhui Chen, Jingmin Zhao, Xiangmei Chen, Weidong Pan, Shuangsuo Dang, Fengmin Lu

**Affiliations:** 1 Department of Microbiology, Infectious Disease Center, School of Basic Medical Sciences, Peking University Health Science Center, Beijing 100191, China; qixijn@bjmu.edu.cn (X.Q.); lauyenna@bjmu.edu.cn (Y.L.); xm_chen6176@bjmu.edu.cn (X.C.); 2 Department of Pancreatic Hepatobiliary Surgery, The Sixth Affiliated Hospital of Sun Yat-sen University, Guangzhou 510655, China; 3 Department of Infectious Diseases, The Second Affiliated Hospital of Xi’an Jiaotong University, Xi’an 710004, China; wfp612526@163.com; 4 Department of Traditional and Western Medical Hepatology, The Third Hospital of Hebei Medical University, Shijiazhuang 050051, China; zhangsiyu9105@163.com (S.Z.); nanyuemin@163.com (Y.N.); 5 Department of Laboratory Medicine, The Third Affiliated Hospital of Sun Yat-sen University, Guangzhou 510630, China; gongjiao@mail2.sysu.edu.cn (J.G.); hubo@mail.sysu.edu.cn (B.H.); 6 Intervention and Cell Therapy Center, Peking University Shenzhen Hospital, Shenzhen 518035, China; chenjunhui@pkuszh.com; 7 Department of Pathology and Hepatology, The 5th Medical Centre, Chinese PLA General Hospital, Beijing 100039, China; jmzhao302@163.com; 8 Precision Medicine Center, Academy of Medical Sciences, Zhengzhou University, Zhengzhou 450001, China; 9 Beijing Key Laboratory of Hepatitis C and Immunotherapy for Liver Diseases, Peking University Hepatology Institute, Peking University People’s Hospital, Beijing 100044, China

**Keywords:** alpha-fetoprotein, hepatitis B virus, antiviral therapy, aspartate aminotransferase, hepatocellular carcinoma

## Abstract

Background and aims: Factors associated with abnormally elevated alpha-fetoprotein (AFP) in hepatitis B virus (HBV)-infected patients remain to be studied. We aimed to identify factors associated with elevated serum AFP in patients with non-hepatocellular carcinoma (HCC) and early-stage HCC and their influences on the performance of AFP for detecting early-stage HCC. Methods: This multicenter, retrospective study was conducted in 4401 patients with chronic HBV infection, including 3680 patients with non-HCC and 721 patients with early-stage HCC. Factors associated with elevated AFP were analyzed. Diagnostic performance of AFP for early-stage HCC were compared among groups through area under the receiver operating characteristic curve (AUC), sensitivity, and specificity. Results: When analyzed by multivariate logistic regression, antiviral therapy was negatively associated with elevated AFP, while hepatitis B e antigen (HBeAg) and aspartate aminotransferase (AST) > 1× upper limit of normal (ULN) were positively associated with elevated AFP both in patients with non-HCC and early-stage HCC (all *p* < 0.05). The AUCs of AFP for detecting early-stage HCC in patients with antiviral therapy, HBV DNA (−), alanine aminotransferase (ALT) ≤ 1× ULN, and AST ≤ 1× ULN were significantly higher compared to those in non-antiviral therapy, HBV DNA (+), ALT > 1× ULN, and AST > 1× ULN groups, respectively. When categorizing patients into AST ≤ 1× ULN and > 1× ULN, AFP achieved the highest AUCs in patients with AST ≤ 1× ULN regardless of antiviral treatment (AUCs = 0.813 and 0.806, respectively). Furthermore, there were considerable differences in the cut-off values of AFP in detecting early-stage HCC in different subgroups when applying similar sensitivity and specificity. Conclusions: Antiviral therapy and serum AST might be used to help judge and select the specific cut-off values of serum AFP for HCC surveillance in different at-risk populations.

## 1. Introduction

Primary liver cancer is the sixth most commonly diagnosed cancer and the third leading cause of cancer death worldwide in 2020, with approximately 906,000 new cases and 830,000 deaths annually, and hepatocellular carcinoma (HCC) accounts for 75–90% cases [1,2]. The major risk factors for HCC are related to the underlying liver diseases and vary from region to region, which is mainly hepatitis B virus (HBV) infection in China [1,3]. Due to the lack of specific symptoms and signs in the early stages of HCC, patients with HCC frequently present with advanced stages once diagnosed and have an extremely poor prognosis, with the 5-year survival rate of only 15~17% as reported [4,5,6,7]. HCC surveillance of high-risk patients is associated with increased early-stage detection and improved opportunity for curative treatment, which was identified as an effective way to improve clinical outcomes and reduce mortality in patients with HCC [2,8,9].

Serum alpha-fetoprotein (AFP) is the most widely used biomarker for the surveillance and auxiliary diagnosis of HCC in real-world clinical practice [10]. However, the utility of serum AFP as a surveillance and detection test of HCC has been seriously challenged because of its suboptimal sensitivity and specificity [11,12,13]. There are many noncancerous factors that have been demonstrated to influence AFP levels and its effectiveness to detect HCC. Serum AFP could be falsely elevated in patients with chronic active hepatitis, advanced fibrosis, and cirrhosis but without evidence of HCC [14,15,16,17]. Previous studies also showed that serum alanine aminotransferase (ALT), aspartate aminotransferase (AST), etiology, and race were associated with elevated AFP in hepatitis C virus (HCV)-infected patients without HCC [14,18,19]. In addition, our previous work revealed that antiviral treatment could lower serum AFP levels in patients with HBV-related liver disease and improve the surveillance performance of serum AFP for early-stage HCC [17]. 

Relatively few data are known about the determinant factors associated with AFP levels in patients with HBV-related chronic liver diseases and HCC. Thus, this study aims to investigate the factors that affect the performance of serum AFP for detecting early-stage HCC. In addition, the optimal cut-off values of AFP for diagnosing HCC under different conditions of antiviral therapy and liver inflammation were studied.

## 2. Materials and Methods

### 2.1. Patients Selection

This retrospective study enrolled 4401 hepatitis B surface antigen (HBsAg)-positive patients from five centers (the Fifth Medical Center of Chinese PLA General Hospital, Beijing; the Third Affiliated Hospital of Sun Yat-Sen University, Guangzhou; Peking University Shenzhen Hospital, Shenzhen; the Third Hospital of Hebei Medical University, Shijiazhuang; the Second Affiliated Hospital of Xi’an Jiaotong University, Xi’an) between 2009 and 2018. The included criteria were as follows: (1) patients were HBsAg-positive for at least 6 months; (2) patients had detailed information on laboratory data including serum AFP test; and (3) patients had clear clinical records of receiving antiviral treatment or not. The exclusion criteria were: (1) patients with liver diseases due to co-infection with HCV or other hepatitis or genetic and autoimmune disorders; (2) HCC patients with prior history of anti-tumor treatment or other malignant tumors; (3) patients with HCC beyond Milan criteria; (4) patients with no insufficient information of relevant laboratory tests and other clinical characteristics; (5) patients with interferon treatment at the time of inclusion; and (6) patients with pregnancy status. A flowchart of patient inclusion is shown in Supplementary Figure S1.

HCC was diagnosed based on histopathological confirmation or detection of a positive lesion with recommended imaging techniques and contrast agents (multiphasic computed tomography and dynamic contrast-enhanced magnetic resonance imaging, contrast-enhanced ultrasonography) [17,20,21]. Early-stage HCC was defined according to the Milan criteria (1 nodule ≤ 5 cm or 2 to 3 nodules with each ≤ 3 cm in diameter, without gross vascular invasion or extrahepatic metastases) [2,22]. Absence of HCC was determined by clinical and imaging evidence lacking any suspicious-appearing hepatic masses in patients with chronic liver disease at enrollment. Patients with an aberrant AFP exceeding normal at enrollment were assessed by a CT or MRI that showed no lesion indicative of HCC within recent months [17]. 

The presence of liver cirrhosis was defined by clinical, laboratory, and imaging features, and liver biopsy was not routinely performed.

### 2.2. Study Variables

Patients who had received continuous antiviral therapy with nucleos(t)ide analogues (NAs) for at least 3 months were categorized as the antiviral group. Patients who were treatment-naïve or had interrupted antiviral therapy for more than 6 months were allocated into the non-antiviral group [17].

Serum AFP levels were measured in local laboratories at each of the five clinical centers by using an automated electrochemiluminescence immunoassay (Roche Diagnostics, USA). The upper limit of normal (ULN) for AFP values among these centers varied between 7.02 and 13.4 ng/mL. The lower and upper limit of detection were the same among centers, with >0 ng/mL and 1210 ng/mL, respectively. The cut-off values of 20 ng/mL, 100 ng/mL, 200 ng/mL, and 400 ng/mL, which were recommended for the investigations and confirmatory tests for HCC [3,17,20,21], were also analyzed to elucidate the clinical utility of AFP.

Serum HBV DNA assays were quantitated through the real-time polymerase chain reaction (PCR) provided by Da’an gene Co., Ltd. (Guangzhou, China) or Fosun Pharmaceutical Co., Ltd. (Shanghai, China). The lower detection of limit was 100 IU/mL for four centers and 200 IU/mL for one center. HBeAg was measured by chemiluminescence immunoassay technique or enzyme-linked immunosorbent assay. Liver-related biochemical testing, routine blood testing, other tests, and the ULNs of serum ALT and AST were determined in local laboratories of each center using commercially available kits. The normal ranges for AFP, ALT, AST, total bilirubin (TBIL), albumin, and platelet counts by each center are shown in Supplementary Table S1. Individual unusual values were reviewed to verify the accuracy of data.

This study was approved by the ethics committee of Peking University Health Science Center. All procedures performed in this study involving human participants were in accordance with the ethical standards of the institutional and/or national research committee and with the 1964 Helsinki Declaration and its later amendments or comparable ethical standards.

### 2.3. Statistical Analysis

Continuous variables are expressed as mean ± standard deviation (SD) or median (first quartile, third quartile) and were compared by *t*-test or Mann–Whitney test between groups. Chi-square test was applied to compare categorical variables. Factors associated with abnormally elevated AFP were tested by constructing univariate and multivariate (forward) logistic regression analysis in non-HCC and early-stage HCC patients. The receiver operating characteristic (ROC) curves were used to analyze the performance of AFP in discriminating HCC from at-risk patients. The area under the ROC curve (AUC) and 95% confidence interval (CI) were also calculated. Sensitivity, specificity, positive likelihood ratio (LR+), and negative likelihood ratio (LR−) of different cut-off values of AFP levels were calculated. All statistical analyses were performed by SPSS 24.0 software (New York, NY, USA), MedCalc version 18.2.1 (MedCalc Software bvba, Ostend, Belgium), and GraphPad Prism version 7.0 (San Diego, CA, USA). All tests of significance were two-tailed, and *p* < 0.05 was considered statistically significant.

## 3. Results

### 3.1. Patient Characteristics

A total of 3680 non-HCC patients with CHB and 721 patients with early-stage HCC were finally enrolled in the study. The mean age of the total cohort was 45.80 ± 12.36 years, and 75.1% were male (*n* = 3305). Clinical and laboratory characteristics of patients between non-antiviral and antiviral groups are summarized in Table 1. Patients in the non-antiviral group, no matter with or without early-stage HCC, had a significantly lower proportion of HBV DNA-negative patients and cirrhotic patients but higher median levels of serum ALT and AST and lower median albumin levels compared to the antiviral group. Noticeably, the proportion of AFP > 1× ULN was also significantly higher in the non-antiviral group than in the antiviral group both for patients with non-HCC (37.3% vs. 11.4%, *p* < 0.001) and early-stage HCC (66.7% vs. 53.6%, *p* < 0.001). The median levels of serum AFP were consistently higher in the non-antiviral than in the antiviral group both in patients with non-HCC (5.58 ng/mL vs. 2.68 ng/mL, *p* < 0.001) and early-stage HCC (29.2 ng/mL vs. 12.1 ng/mL, *p* < 0.001). Furthermore, a larger tumor size was found in HCC patients of the non-antiviral group when compared to that of the antiviral group (*p* < 0.001).

### 3.2. Factors Independently Associated with Abnormally Elevated AFP (>1× ULN) in Non-HCC and Early-Stage HCC Patients

The univariate and multivariate logistic regression analyses were applied to identify the independent factors associated with abnormally elevated AFP (>1× ULN) in non-HCC and early-stage HCC patients. As shown in Table 2, for non-HCC patients when analyzed by multivariate logistic regression, antiviral therapy (OR: 0.772, 95%CI: 0.601–0.993, *p* = 0.044) and albumin (OR: 0.960, 95%CI: 0.945–0.976, *p* < 0.001) were independently negatively associated with elevated AFP; HBeAg (OR: 1.353, 95%CI: 1.115–1.641, *p* = 0.002), HBV DNA (OR: 2.860, 95%CI: 2.164–3.780, *p* < 0.001), cirrhosis (OR: 1.456, 95%CI: 1.151–1.841, *p* = 0.002), ALT > 1× ULN (OR: 2.051, 95%CI: 1.585–2.656, *p* < 0.001), AST > 1× ULN (OR: 2.655, 95%CI: 2.015–3.499, *p* < 0.001), and TBIL (OR: 1.006, 95%CI: 1.004–1.007, *p* < 0.001) were independently positively associated with elevated AFP.

As shown in Table 3, for early-stage HCC patients when analyzed by multivariate logistic regression, antiviral therapy (OR: 0.632, 95%CI: 0.463–0.865, *p* = 0.004) and gender (OR: 0.549, 95%CI: 0.358–0.842, *p* = 0.006) were independently negatively associated with elevated AFP; HBeAg (OR: 1.535, 95%CI: 1.080–2.182, *p* = 0.017) and AST > 1× ULN (OR: 1.780, 95%CI: 1.262–2.510, *p* = 0.001) were independently positively associated with elevated AFP (OR > 1, *p* < 0.05).

The above results demonstrate that whether in non-HCC or in early-stage HCC patients, antiviral therapy, HBeAg, and AST were independently associated with abnormally elevated AFP. Notably, some additional factors showed a significant association with elevated AFP by univariate analysis, such as gender and platelet in non-HCC patients and HBV DNA, ALT > 1× ULN, and TBIL in patients with early-stage HCC.

Furthermore, the median values of AFP in the antiviral group were significantly lower compared to the non-antiviral group in each of HBeAg, HBV DNA, ALT, and AST subgroups for non-HCC patients (Supplementary Figure S2). Similar results were observed in early-stage HCC patients (Supplementary Figure S3).

### 3.3. Performance of Serum AFP in Discriminating Early-Stage HCC in Different Subgroups

**Figure 1:**
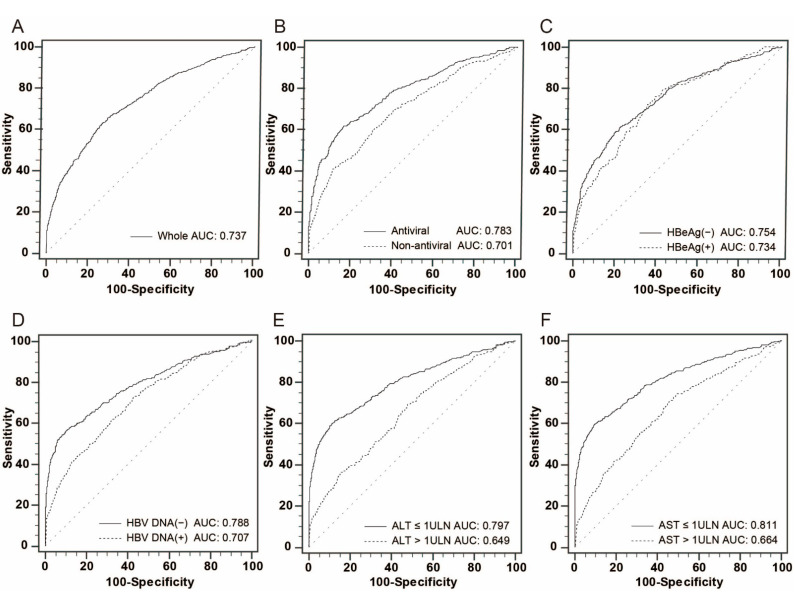
ROC curves of serum AFP discriminating early-stage HCC in different subgroups. (**A**) The whole cohort. (**B**) Between antiviral and non-antiviral subgroups. (**C**) Between HBeAg (−) and HBeAg (+) subgroups. (**D**) Between HBV DNA (−) and HBV DNA (+) subgroups. (**E**) Between ALT ≤ 1× ULN and ALT > 1× ULN subgroups. (**F**) Between AST ≤ 1× ULN and AST > 1× ULN subgroups.

As antiviral therapy was negatively associated and HBeAg and AST > 1× ULN were positively associated with elevated AFP in patients with both non-HCC and early-stage HCC, we then analyzed the AUCs of AFP in discriminating early-stage HCC with patients divided by antiviral therapy, HBeAg, HBV DNA, ALT, and AST. As shown in Figure 1, the AUC of AFP was significantly higher in the antiviral group compared with the non-antiviral group for detecting early-stage HCC (0.783 vs. 0.701, *p* < 0.001). Similar results were observed in the subgroups of HBV DNA (−) and HBV DNA (+) (0.788 vs. 0.707, *p* < 0.001), ALT ≤ 1× ULN and ALT > 1× ULN (0.797 vs. 0.649, *p* < 0.001), and AST ≤ 1× ULN and AST > 1× ULN (0.811 vs. 0.664, *p* < 0.001) except for the subgroups between HBeAg (−) and HBeAg (+) (0.754 vs. 0.734, *p* = 0.397). Interestingly, the higher AUC values always occurred in the subgroups of patients with lower HBV virus replication and less inflammation and damage of the liver, such as the subgroups of antiviral group, HBeAg (−), HBV DNA (−), ALT ≤ 1× ULN, and AST ≤ 1× ULN, as well as the lower optimal cut-off values compared to the corresponding subgroups. Supplementary Table S2 also shows the corresponding cutoffs, LR+ and LR−, for AFP determined by the point in the ROC curve that maximizes sensitivity and specificity.

### 3.4. Better Performance of Serum AFP in Discriminating Early-Stage HCC at the Subgroup of AST ≤ 1× ULN Both in Antiviral and Non-Antiviral Groups

As is well-known, antiviral treatment has a remarkable influence on the level of HBV replication, leading to the suppression of HBV DNA and HBeAg loss and occasionally to HBeAg loss and seroconversion to anti-HBe, which further have been shown to achieve the elimination of HBV-induced necroinflammatory activity. Then, as shown in Table 4, the AUCs of AFP for each subgroup of patients divided by HBeAg, HBV DNA, ALT, and AST were analyzed and compared between the respective non-antiviral and antiviral groups. In different subgroups of antiviral therapy, the AUC of AFP in discriminating early-stage HCC was significantly higher in patients with ALT ≤ 1× ULN compared to patients with ALT > 1× ULN (0.796 vs. 0.682, *p* < 0.01) and as well as for patients with AST ≤ 1× ULN compared to patients with AST > 1× ULN (0.806 vs. 0.711, *p* < 0.01). As for the subgroups of non-antiviral therapy, similar trends were also observed for ALT ≤ 1× ULN compared to ALT > 1× ULN (0.788 vs. 0.667, *p* < 0.001) and AST ≤ 1× ULN compared to AST > 1× ULN (0.813 vs. 0.669, *p* < 0.001). Although there were no significant differences, the AUCs were marginally higher in the HBV DNA (−) subgroup compared with the HBV DNA (+) subgroup both in the non-antiviral and antiviral groups (0.767 vs. 0.700, *p* = 0.091 and 0.791 vs. 0.748, *p* = 0.307, respectively). Interestingly, the best discriminating performance of AFP was achieved in the subgroup of AST ≤ 1× ULN both in the non-antiviral and antiviral groups.

### 3.5. The Influence of Antiviral Therapy and Serum AST on the Cut-Off Values of Serum AFP in Discriminating Early-Stage HCC

Firstly, we analyzed the proportions of abnormal AFP levels between AST ≤ 1× ULN and AST > 1× ULN in patients with different liver diseases in non-antiviral and antiviral groups (Supplementary Figure S4). For non-antiviral group, the abnormal percentage of AFP levels were lower in patients with AST ≤ 1× ULN than in patients with AST > 1× ULN in the CHB, cirrhosis, and early-stage HCC subgroups (8.7% vs. 46.0%, *p* < 0.001; 15.6% vs. 58.2%, *p* < 0.001; and 58.1% vs. 76.1%, *p* < 0.001, respectively). Similarly, for the antiviral group, patients with AST ≤ 1× ULN had significantly lower percentage of abnormal AFP levels compared to those with AST > 1× ULN in CHB, cirrhosis, and early-stage HCC subgroups (4.0% vs. 21.8%, *p* < 0.001; 5.5% vs. 32.7%, *p* < 0.001; and 48.4% vs. 64.5%, *p* = 0.004, respectively).

Then, the influence on the cut-off values of AFP by antiviral therapy and serum AST were further analyzed (Table 5). As shown in Table 5, whether in the non-antiviral group or in antiviral group, the specificities of AFP in AST ≤ 1× ULN were always remarkably higher than those in AST > 1× ULN at the cut-off values of ULN and 20 ng/mL, partly compromising the corresponding sensitivities. At the same time, the cut-off values of ULN and 20 ng/mL had higher sensitivities compared to 100 ng/mL, 200 ng/mL, and 400 ng/mL in patients with AST ≤ 1× ULN of the non-antiviral group, from 58.10% and 50.84% to 34.64%, 25.14%, and 16.76%, and the corresponding specificity and LR+ remained in high levels. However, in patients with AST > 1× ULN of the non-antiviral group, the specificities of ULN and 20 ng/mL greatly declined as the cut-off values changed to 100 ng/mL, 200 ng/mL, and 400 ng/mL, and the LR+ remained low. In addition, the above same tendencies of these changes were also observed between AST ≤ 1× ULN and AST > 1× ULN in the antiviral group (Table 5).

Furthermore, cut-off values of AFP in antiviral group were obviously lower compared to those in the non-antiviral group when considering the similar sensitivity and specificity. For example, the sensitivity and specificity of ULN in AST ≤ 1× ULN of the antiviral group were 48.45% and 95.26%, but in AST ≤ 1× ULN of the non-antiviral group, the cut-off value of AFP was 20 ng/mL considering the similar sensitivity (50.84%) and specificity (94.90%) as well as the similar trend in AST > 1× ULN between antiviral and non-antiviral groups.

## 4. Discussion

In this large multi-center study, we simultaneously incorporated the antiviral therapy, virological, and inflammatory variables of the liver into the analysis of determinant factors associated with abnormally elevated serum AFP levels in patients with HBV infection. Antiviral therapy was negatively associated with abnormally elevated AFP, while HBeAg and AST > 1× ULN were positively associated both in patients with non-HCC and early-stage HCC. The AUCs of subgroups of antiviral therapy, namely HBV DNA (−), ALT ≤ 1× ULN, and AST ≤ 1× ULN, were significantly higher compared to the corresponding subgroups, which were also observed in the subgroups of patients with lower virus activation and less inflammation and damage of the liver. Serum AFP in the subgroup of AST ≤ 1× ULN always had the best discriminating performance both in the non-antiviral and antiviral groups. Further analysis also showed that there were considerable differences of the cut-off values of AFP discriminating early-stage HCC in different subgroups when considering the similar sensitivity and specificity, for example, the ULN in AST ≤ 1× ULN of antiviral and 20 ng/mL in AST ≤ 1× ULN of the non-antiviral groups.

Different from the European and American guidelines suggesting surveillance using ultrasonography with or without AFP every 6 months [2,8], the surveillance strategy of Asia-Pacific and China suggests the combined use of U.S. and serum AFP measurement biannually [3,21]. In addition, serum AFP is also an auxiliary test in combination with imaging for the diagnosis of HCC in China [21]. A meta-analysis study comparing the accuracy of ultrasound with or without AFP for HCC surveillance found that ultrasound with AFP had a significantly higher sensitivity than ultrasound alone, with the pooled sensitivity of 63% (95%CI, 48–75%) and 45% (95%CI, 30–62%) for early-stage HCC, respectively (*p* = 0.002), which suggests that, among currently available tests, ultrasound in combination with AFP may be the most effective strategy for HCC surveillance in patients with cirrhosis [10,23]. Despite some controversies, serum AFP measurement is still broadly employed as a conventional and relatively highly effective promising biomarker for surveillance and auxiliary diagnosis of HCC in real-world clinical settings [23,24].

Several factors have been identified to be associated with elevated serum AFP in patients with liver disease of chronic hepatitis C other than HCC, such as elevated ALT, decreased platelet count, lower albumin levels, older age, female gender, ethnicity, and advanced fibrosis [14,19,25,26,27]. Our present study showed that factors associated with abnormally elevated AFP in non-HCC were predominantly reflecting the status of viral replication of HBV, inflammatory damage, and function (e.g., with antiviral therapy, HBV DNA (−), ALT > 1× ULN, AST > 1× ULN, cirrhosis, albumin, and TBIL levels) in liver. Besides, partly different from the previous study of patients with HCV-related HCC, i.e., that gender, race, and serum ALT were independently associated with AFP ≥ 20 ng/mL [19], we found that antiviral therapy, HBeAg, AST > 1× ULN, and gender were independent factors associated with abnormally elevated AFP in HBV-related early-stage HCC patients. However, the potential mechanisms of the associations between elevated AFP and these factors remained to be limited understanding. One likely explanation is that the presence of enhanced hepatocyte destruction and regeneration of liver progenitor cells with a less differentiated phenotype induced by massive liver damage, including severe inflammation, fibrosis, and bridging hepatic necrosis, lead to AFP production [17,27,28,29]. Additionally, HBV viral transcription co-regulator HBx could directly transcriptionally upregulate AFP gene expression [30]. Moreover, ALT is exclusively located in the cytoplasm and AST mainly located in the mitochondria of hepatocytes, and the AST level will exceed the ALT level when released from mitochondrial AST compartments as a consequence of more severe liver damage [31,32].

It has been shown that the AUC of serum AFP for HCC was significantly higher in HCV patients with ALT ≤ 40 U/L than patients with ALT > 40 U/L but not in HBV patients [19], and antiviral therapy improved the AUC of AFP for diagnosing early-stage HCC rather than late-stage HCC in our previous study [17]. Except for ALT and antiviral therapy, herein, our analysis showed that HBV DNA and AST also had significant influence on the performance of serum AFP for detecting early-stage HCC. Patients with the lower HBV virus replication and less inflammation and damage of the liver (e.g., with antiviral therapy, HBV DNA (−), ALT ≤ 1× ULN, and AST ≤ 1× ULN) always had significantly higher AUCs compared to the corresponding subgroups. After controlling the influence of antiviral treatment on HBV replication and liver inflammation, the above similar results were also observed for each subgroup regardless of whether in the non-antiviral or antiviral groups. Further, the AUCs of AFP for early-stage HCC in AST ≤ 1× ULN were marginally higher than those in ALT ≤ 1× ULN, but there were no significant differences as well as between AST > 1× ULN and ALT > 1× ULN regardless of whether in the whole group, non-antiviral group, or antiviral group. Additionally, the highest AUCs of AFP for discriminating early-stage HCC were both seen in the subgroup of AST ≤ 1× ULN. These results suggest that antiviral therapy and serum AST might be the two considerably important factors influencing the performance of serum AFP for detecting early-stage HCC compared to other factors.

The newest Asia-Pacific guidelines have recommended that the cut-off value of AFP can be set at lower value than 200 ng/mL in a population with hepatitis virus suppression or eradication; however, there were no specific cut-off values to recommend reference [3,33,34]. In the present study, going deeper into the analysis of the influence on the cut-off values of AFP by antiviral therapy and AST, the results showed that different subgroups of patients might refer to different cut-off values when considering the similar sensitivity and specificity. For patients with antiviral therapy, ULN and 100 ng/mL might be the optimal cut-offs of AFP for detecting early-stage HCC in patients with AST ≤ 1× ULN and AST > 1× ULN, respectively, while in patients without antiviral therapy, the corresponding cut-offs might be 20 ng/mL and 200 ng/mL, respectively. At the same time, 20 ng/mL and 100 ng/mL might be also considered for the surveillance of early-stage HCC due to the remarkably increased sensitivity and acceptable specificity compared to 100 ng/mL and 200 ng/mL in patients of antiviral and non-antiviral group with AST > 1× ULN, respectively. Hence, different cut-off values of AFP for detecting HCC should be referred in populations with a different status of HBV suppression and liver inflammation, and receiving antiviral therapy and normal of AST would be the two important and referable indicators to conduct a judgement and selection on the specific cut-off values, which appears to be particularly important, as more and more patients with HBV infection receive highly potent NAs antiviral therapy.

Our study has several limitations. This is a retrospective study; although it includes five tertiary centers and a large-scale number of patients, and all data adhere to the same quality control specification, which would increase the statistical power and reliability of results, some unmeasured potential biases might also exist. The results of this study might not expand to other liver diseases because of its study population with only HBV infection. In addition, the association between ALT and the cut-off values of AFP for early-stage HCC were not further evaluated because the influence of AST on the performance of AFP for early-stage HCC seems to be more significant, and the elevation of AST level represents the more severe damage of the liver, which generally changes in parallel with ALT.

In conclusion, our study suggests that in patients with chronic HBV infection, antiviral therapy, HBeAg, and AST > ULN were independently associated with abnormally elevated AFP both in patients with non-HCC and early-stage HCC. Antiviral therapy and serum AST would apparently affect the performance and cut-off values of serum AFP for detecting early-stage HCC and would be the two important and referable indicators when judging and selecting the specific cut-off values for HCC surveillance in different at-risk populations with different statuses of HBV suppression and liver inflammation.

## Figures and Tables

**Table 1:** Clinical characteristics of patients with non-HCC and early-stage HCC.

Variable	Non-HCC			Early-Stage HCC		
	Non-Antiviral (*n* = 1803)	Antiviral (*n* = 1877)	*p*-Value	Non-Antiviral (*n* = 342)	Antiviral (*n* = 379)	*p*-Value
Age (year)	42.58 ± 12.78	46.07 ± 11.44	<0.001	53.59 ± 10.29	52.73 ± 10.11	0.260
Male, *n* (%)	1337 (74.2)	1375 (73.3)	0.536	282 (82.5)	311 (82.1)	0.889
HBeAg (+/−)	845/958	579/1298	<0.001	104/238	104/275	0.380
HBV DNA (+/−)	1540/263	333/1544	<0.001	268/74	75/304	<0.001
Cirrhosis (+/−)	793/1010	1069/808	<0.001	293/49	350/29	0.004
ALT (IU/L)	60 [30, 301]	25 [18, 36]	<0.001	38 [25, 57]	29 [21, 42]	<0.001
ALT > 1× ULN *, *n* (%)	1123 (62.3)	337 (18.0)	<0.001	149 (43.6)	105 (27.7)	<0.001
AST (IU/L)	58 [31, 176]	28 [22, 40]	<0.001	39 [28, 64]	31 [24, 45]	<0.001
AST > 1× ULN *, *n* (%)	1156 (64.1)	486 (25.9)	<0.001	163 (47.7)	121 (31.9)	<0.001
TBIL (μmol/L)	21.2 [13.5, 44.8]	15.3 [11.2, 23.2]	<0.001	17.7 [12.0, 26.6]	16.4 [12.0, 24.4]	0.221
Albumin (g/L)	39.3 [33.0, 43.8]	41.5 [36.0, 45.2]	<0.001	38.0 [33.2, 41.0]	40.0 [35.0, 43.0]	<0.001
Platelet (×10^9^/L)	149 [91, 202]	136 [78, 191]	<0.001	113 [72, 170]	107 [71, 154]	0.055
Tumor size (cm)	-	-	-	2.86 ± 1.16	2.51 ± 1.10	<0.001
Number of tumors (1/2–3)	-	-	-	314/28	342/37	0.461
AFP (ng/mL)	5.58 [2.61, 25.4]	2.68 [1.75, 4.60]	<0.001	29.2 [5.38, 275.1]	12.1 [3.57, 125.5]	<0.001
AFP > 1× ULN *, *n* (%)	672 (37.3)	213 (11.4)	<0.001	228 (66.7)	203 (53.6)	<0.001

HCC, hepatocellular carcinoma; HBeAg, hepatitis B e antigen; ALT, alanine aminotransferase; AST, aspartate aminotransferase; TBIL, total bilirubin; AFP, alpha-fetoprotein; ULN, upper limit of normal. Continuous variables are expressed as mean ± SD or median [first quartile, third quartile]. * The ULN of ALT, AST, and AFP values are based on the various ULN for the five clinical centers.

**Table 2:** Factors associated with abnormally elevated AFP (>1× ULN) by univariate and multivariate logistic analysis in non-HCC patients.

Non-HCC	Univariate		Multivariate	
	OR (95%CI)	*p*-Value	OR (95%CI)	*p*-Value
Age (year)	0.996 (0.990, 1.002)	0.239	-	-
Gender (M)	1.287 (1.077, 1.538)	0.005	-	-
HBeAg (+)	2.226 (1.909, 2.595)	<0.001	1.353 (1.115, 1.641)	0.002
HBV DNA (+)	7.486 (6.170, 9.083)	<0.001	2.860 (2.164, 3.780)	<0.001
Cirrhosis (+)	1.581 (1.356, 1.842)	<0.001	1.456 (1.151, 1.841)	0.002
ALT > 1× ULN	7.972 (6.695, 9.492)	<0.001	2.051 (1.585, 2.656)	<0.001
AST > 1× ULN	11.491 (9.425, 14.011)	<0.001	2.655 (2.015, 3.499)	<0.001
TBIL (μmol/L)	1.014 (1.012, 1.015)	<0.001	1.006 (1.004, 1.007)	<0.001
Albumin (g/L)	0.908 (0.898, 0.918)	<0.001	0.960 (0.945, 0.976)	<0.001
Platelet (×10^9^/L)	0.996 (0.995, 0.997)	<0.001	-	-
Antiviral therapy (+)	0.215 (0.181, 0.256)	<0.001	0.772 (0.601, 0.993)	0.044

HCC, hepatocellular carcinoma; M, male; HBeAg, hepatitis B e antigen; AFP, alpha-fetoprotein; OR, odds ratio; ALT, alanine aminotransferase; AST, aspartate aminotransferase; TBIL, total bilirubin; ULN, upper limit of normal.

**Table 3:** Factors associated with abnormally elevated AFP (>1× ULN) by univariate and multivariate logistic analysis in early-stage HCC patients.

Early-Stage HCC	Univariate		Multivariate	
	OR (95%CI)	*p*-Value	OR (95%CI)	*p*-Value
Age (year)	0.997 (0.983, 1.012)	0.713	-	-
Gender (M)	0.546 (0.361, 0.828)	0.004	0.549 (0.358, 0.842)	0.006
HBeAg (+)	1.668 (1.187, 2.343)	0.003	1.535 (1.080, 2.182)	0.017
HBV DNA(+)	1.880 (1.388, 2.546)	<0.001	-	-
Cirrhosis (+)	1.562 (0.975, 2.502)	0.064	-	-
ALT > 1× ULN	1.556 (1.132, 2.139)	0.007	-	-
AST > 1× ULN	2.238 (1.628, 3.075)	<0.001	1.780 (1.262, 2.510)	0.001
TBIL (μmol/L)	1.010 (1.003, 1.017)	0.006	-	-
Albumin (g/L)	0.976 (0.952, 1.000)	0.055	-	-
Platelet (×10^9^/L)	0.998 (0.996, 1.001)	0.134	-	-
Tumor size (cm)	1.038 (0.911, 1.183)	0.577	-	-
Number of tumors (2–3/1)	1.253 (0.736, 2.133)	0.405	-	-
Antiviral therapy (+)	0.577 (0.426, 0.780)	<0.001	0.632 (0.463, 0.865)	0.004

HCC, hepatocellular carcinoma; M, male; HBeAg, hepatitis B e antigen; AFP, alpha-fetoprotein; OR, odds ratio; ALT, alanine aminotransferase; AST, aspartate aminotransferase; TBIL, total bilirubin; ULN, upper limit of normal.

**Table 4:** Performance characteristics of serum AFP discriminating early-stage HCC in different subgroups for non-antiviral and antiviral groups.

Subgroups of Non-Antiviral Therapy (*n* = 2145)							
Variables	AUC (95%CI)	Cut-Off	Se (%)	Sp (%)	LR+	LR−	*p*-Value
HBeAg							0.976
−	0.720 (0.682, 0.758)	6.90	68.07	65.45	1.97	0.49	-
+	0.719 (0.665, 0.773)	10.14	77.88	54.56	1.71	0.41	-
HBV DNA							0.091
−	0.767 (0.698, 0.836)	10.45	56.76	87.83	4.66	0.49	-
+	0.700 (0.665, 0.736)	87.40	43.28	86.62	3.24	0.65	-
ALT							<0.001
≤1× ULN	0.788 (0.747, 0.829)	15.11	59.59	88.97	5.40	0.45	-
>1× ULN	0.667 (0.619, 0.715)	87.40	44.30	82.99	2.60	0.67	-
AST							<0.001
≤1× ULN	0.813 (0.773, 0.854)	6.90	64.80	85.94	4.61	0.41	-
>1× ULN	0.669 (0.621, 0.716)	87.40	44.79	82.35	2.54	0.67	-
**Subgroups of Antiviral Therapy (*n* = 2256)**							
**Variables**	**AUC (95%CI)**	**Cut-Off**	**Se (%)**	**Sp (%)**	**LR+**	**LR−**	***p*-Value**
HBeAg							0.525
−	0.780 (0.746, 0.813)	7.02	55.64	88.06	4.66	0.50	-
+	0.801 (0.752, 0.850)	6.39	74.04	79.97	3.70	0.32	-
HBV DNA							0.307
−	0.791 (0.759, 0.822)	7.04	55.92	90.93	6.17	0.48	-
+	0.748 (0.689, 0.808)	6.40	78.67	62.16	2.08	0.34	-
ALT							0.001
≤1× ULN	0.796 (0.763, 0.829)	7.32	56.57	91.82	6.91	0.47	-
>1× ULN	0.682 (0.624, 0.739)	6.10	71.43	60.24	1.80	0.47	-
AST							0.002
≤1× ULN	0.806 (0.773, 0.839)	6.56	56.20	92.52	7.52	0.47	-
>1× ULN	0.711 (0.657, 0.764)	10.80	62.81	73.66	2.38	0.50	-

AUC, area under the ROC curve; CI, confidence interval; Se, sensitivity; Sp, specificity; LR+, positive likelihood ratio; LR−, negative likelihood ratio; HBeAg, hepatitis B e antigen; ALT, alanine aminotransferase; AST, aspartate aminotransferase; ULN, upper limit of normal.

**Table 5:** Sensitivity, specificity, LR+, and LR− of different AFP levels for the detection of early-stage HCC between AST ≤ 1× ULN and AST > 1× ULN subgroups in non-antiviral and antiviral groups.

Non-Antiviral Group (*n* = 2145)								
AFP, ng/mL	AST ≤ 1× ULN				AST > 1× ULN			
	Se (%)	Sp (%)	LR+	LR−	Se (%)	Sp (%)	LR+	LR−
ULN	58.10	88.87	5.22	0.47	76.07	48.10	1.47	0.50
20	50.84	94.90	9.97	0.52	61.35	60.21	1.54	0.64
100	34.64	99.07	37.35	0.66	41.72	83.74	2.57	0.70
200	25.14	99.38	40.66	0.75	31.90	89.97	3.18	0.76
400	16.76	99.69	54.22	0.83	19.02	95.42	4.15	0.85
**Antiviral Group (*n* = 2256)**								
**AFP, ng/mL**	**AST ≤ 1× ULN**				**AST > 1× ULN**			
	**Se (%)**	**Sp (%)**	**LR+**	**LR−**	**Se (%)**	**Sp (%)**	**LR+**	**LR−**
ULN	48.45	95.26	10.21	0.54	64.46	69.75	2.13	0.51
20	41.09	98.63	30.08	0.60	50.41	81.07	2.66	0.61
100	27.91	99.86	194.09	0.72	26.45	93.00	3.78	0.79
200	22.48	99.93	312.71	0.78	15.70	95.88	3.82	0.88
400	15.89	100	-	0.84	12.40	97.94	6.02	0.89

AFP, alpha-fetoprotein; Se, sensitivity; Sp, specificity; LR+, positive likelihood ratio; LR−, negative likelihood ratio; ULN, upper limit of normal.

## Data Availability

The datasets analyzed during the current study are available from the corresponding author on reasonable request.

## Supplementary Materials

The following supporting information can be downloaded at: https://www.mdpi.com/article/10.3390/v14081669/s1, Figure S1: The distribution and analysis process of patients enrolled in this study, Figure S2: Comparisons of serum AFP levels between non-HCC patients with and without antiviral treatment in different subgroups, Figure S3: Comparisons of serum AFP levels between early stage HCC patients with and without antiviral treatment in different subgroups, Figure S4: Proportions of abnormal AFP levels between AST ≤ 1×ULN and AST > 1×ULN for patients with chronic hepatitis B (CHB), liver cirrhosis (LC) and early stage HCC in (A) the non-antiviral therapy group, (B) the antiviral therapy group, Table S1: The normal ranges for AFP, ALT, AST, TBIL, Albumin and Platelet by each clinical center, Table S2: Performance characteristics of serum AFP discriminating early stage HCC in different subgroups.

## Abbreviations

AFP	alpha-fetoprotein
HCC	hepatocellular carcinoma
CHB	chronic hepatitis B
HBeAg	hepatitis B e antigen
NAs	nucleos(t)ide analogues
SD	standard deviation
IQR	inter quartile range
ALT	alanine aminotransferase
AST	aspartate aminotransferase
TBIL	total bilirubin
ROC curve	receiver operating characteristic curve
AUC	area under the ROC curve
OR	odds ratio
CI	confidence interval
LR+	positive likelihood ratio
LR−	negative likelihood ratio
ULN	upper limit of normal
