# Supplementary material for: The Performance of Serum Alpha-Fetoprotein for Detecting Early-Stage Hepatocellular Carcinoma Is Influenced by Antiviral Therapy and Serum Aspartate Aminotransferase: A Study in a Large Cohort of Hepatitis B Virus-Infected Patients
Source: Viruses. 2022 Jul 29;14(8):1669. doi: 10.3390/v14081669 (PMC9416230; doi:10.3390/v14081669)
Supplement: Supplementary file 1 [file viruses-14-01669-s001.zip › viruses-1718431-supplementary.pdf]

Supplementary Materials

Supplementary Figures

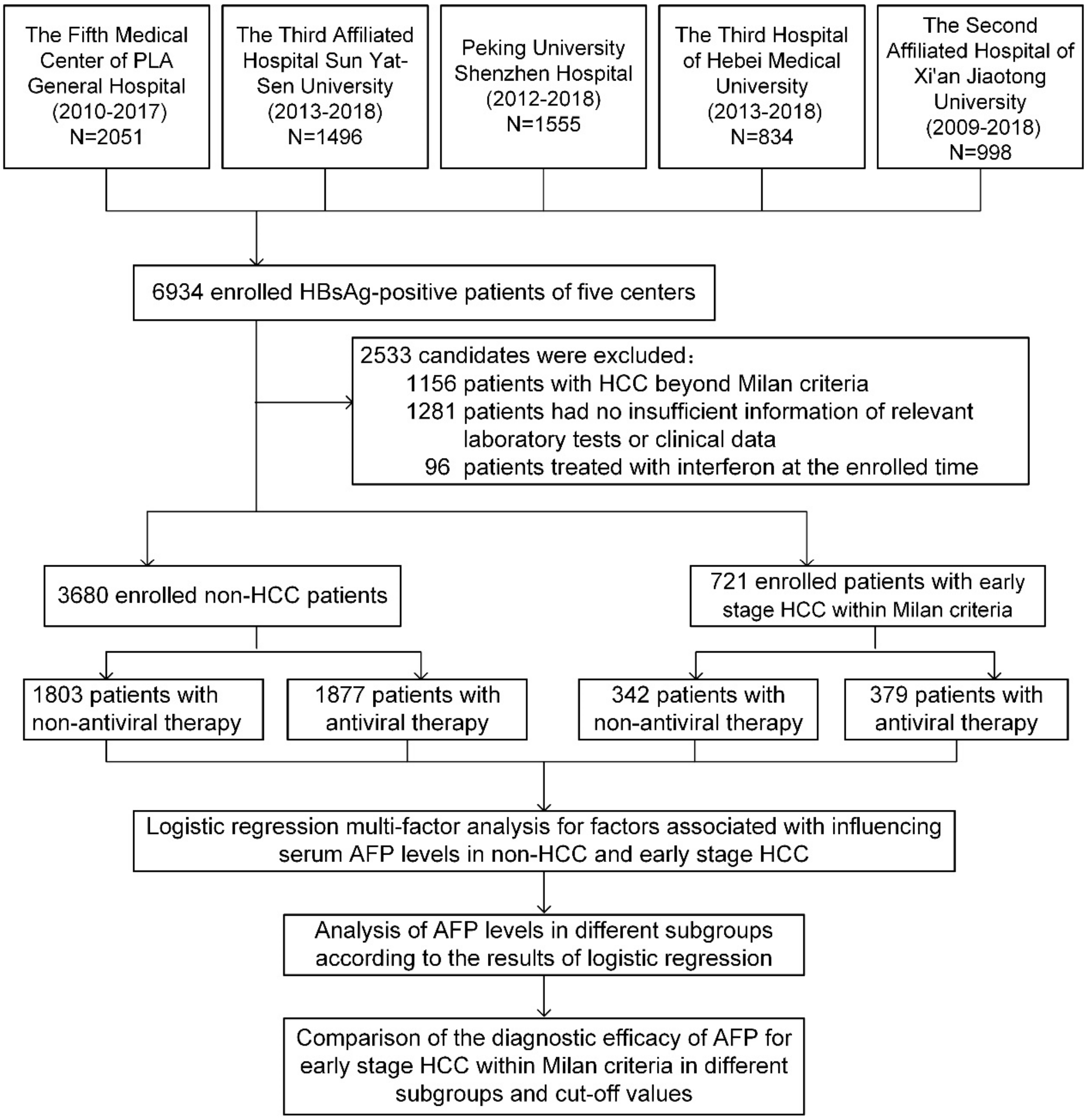

Supplementary Figure S1. The distribution and analysis process of patients enrolled in this study.

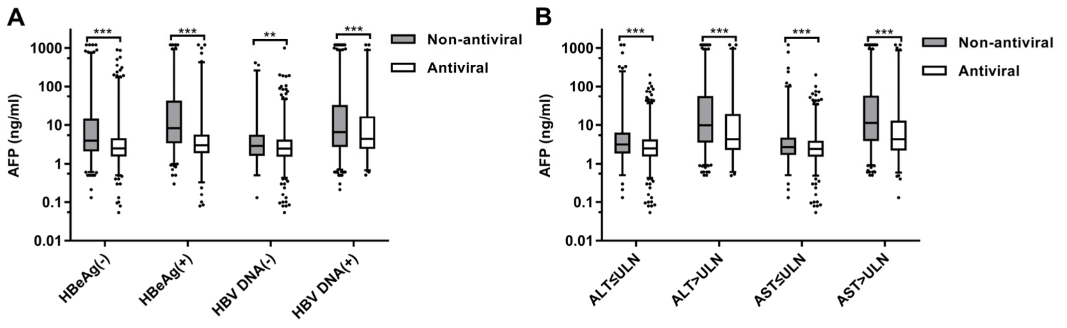

Supplementary Figure S2. Comparisons of serum AFP levels between non-HCC patients with and without antiviral treatment in different subgroups. (A) Subgroups of HBeAg(–), HBeAg(+), HBV DNA (–) and HBV DNA(+); (B) Subgroups of ALT ≤ 1×ULN, ALT > 1×ULN, AST ≤ 1×ULN and AST > 1×ULN. *****P* < 0.01, ******P* < 0.001.

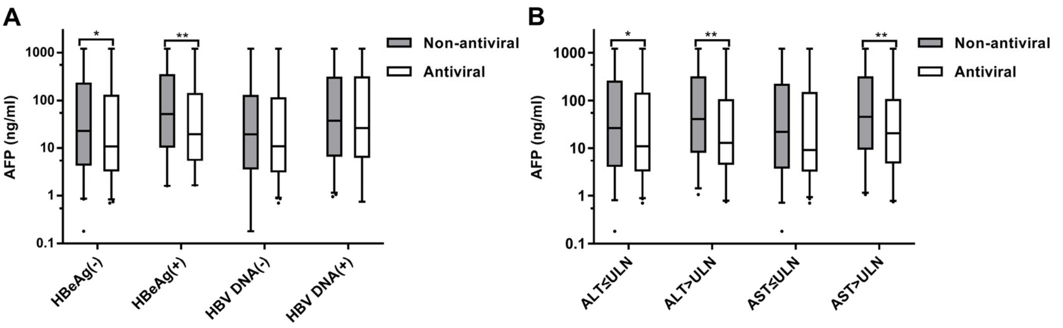

Supplementary Figure S3. Comparisons of serum AFP levels between early stage HCC patients with and without antiviral treatment in different subgroups. (A) Subgroups of HBeAg (–), HBeAg (+), HBV DNA (–) and HBV DNA (+); (B) Subgroups of ALT ≤ 1×ULN, ALT > 1×ULN, AST ≤ 1×ULN and AST > 1×ULN. *P < 0.05, **P < 0.01.

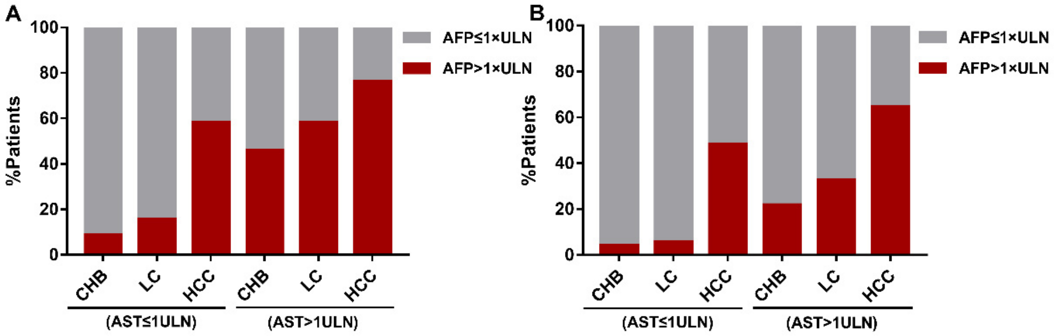

Supplementary Figure S4. Proportions of abnormal AFP levels between AST ≤ 1×ULN and AST > 1×ULN for patients with chronic hepatitis B (CHB), liver cirrhosis (LC) and early stage HCC in (A) the non-antiviral therapy group, (B) the antiviral therapy group.

Supplementary Table S1 The normal ranges for AFP, ALT, AST, TBIL, Albumin and Platelet by each clinical center.

Variables	Center A	Center B	Center C	Center D	Center E
AFP(ng/mL)	0~10	0~8.1	0~13.4	0~9	0~7.02
ALT(U/L)					
Male	5~40	3~35	9~50	9~50	9~50
Female	5~35	3~35	7~40	7~40	7~40
AST(U/L)					
Male	8~40	15~40	15~40	15~40	15~40
Female	8~40	13~35	13~35	13~35	13~35
TBIL(μmol/L)	3.4~20.5	4~23.9	6.1~25.9	3~20	5-20
Albumin (g/L)	35~55	36~51	40~55	40~55	35~55
Platelet($10^{\wedge 9}$/L)	85~303	100~350	125~350	125~350	100~300

Center A: The Fifth Medical Center of Chinese PLA General Hospital; Center B: The Third Affiliated Hospital of Sun Yat-Sen University; Center C: Peking University Shenzhen Hospital; Center D: The Third Hospital of Hebei Medical University; Center E: The Second Affiliated Hospital of Xi'an Jiaotong University; AFP, alpha-fetoprotein; ALT, alanine aminotransferase; AST, aspartate aminotransferase; TBIL, total bilirubin.

Supplementary Table S2. Performance characteristics of serum AFP discriminating early stage HCC in different subgroups.

Variables	AUC (95%CI)	Cut-off	Se (%)	Sp (%)	LR+	LR−	*P*-value
Whole cohort	0.737 (0.716, 0.758)	8.30	62.69	74.37	2.45	0.50	-
Antiviral Therapy							< 0.001
+	0.783 (0.756, 0.811)	6.33	61.74	84.02	3.86	0.46	-
−	0.701 (0.669, 0.733)	16.07	59.65	70.22	2.00	0.57	-
HBeAg							0.397
−	0.754 (0.729, 0.779)	6.90	61.40	78.37	2.84	0.49	-
+	0.734 (0.697, 0.771)	7.38	75.96	61.45	1.97	0.39	-
HBV DNA							< 0.001
−	0.788 (0.759, 0.816)	7.04	56.88	89.60	5.47	0.48	-
+	0.707 (0.677, 0.738)	7.99	72.59	57.29	1.70	0.48	-
ALT							< 0.001
≤ 1×ULN	0.797 (0.772, 0.823)	7.73	59.96	88.83	5.37	0.45	-
> 1×ULN	0.649 (0.612, 0.686)	8.60	69.69	51.85	1.45	0.58	-
AST							< 0.001
≤ 1×ULN	0.811 (0.786, 0.837)	6.90	59.27	90.78	6.42	0.45	-
> 1×ULN	0.664 (0.628, 0.699)	10.14	70.07	55.18	1.56	0.54	-

AUC, area under the ROC curve; CI, confidence interval; Se, sensitivity; Sp, specificity; LR+, positive likelihood ratio; LR−, negative likelihood ratio; HBeAg, hepatitis B e antigen; ALT, alanine aminotransferase; AST, aspartate aminotransferase; ULN, upper limit of normal.
